# Deep learning to assess microsatellite instability directly from histopathological whole slide images in endometrial cancer

**DOI:** 10.1038/s41746-024-01131-7

**Published:** 2024-05-29

**Authors:** Ching-Wei Wang, Hikam Muzakky, Nabila Puspita Firdi, Tzu-Chien Liu, Po-Jen Lai, Yu-Chi Wang, Mu-Hsien Yu, Tai-Kuang Chao

**Affiliations:** 1 Graduate Institute of Biomedical Engineering, National Taiwan University of Science and Technology, Taipei, Taiwan; 2 Department of Gynecology and Obstetrics, Tri-Service General Hospital, Taipei, Taiwan; 3 Department of Gynecology and Obstetrics, National Defense Medical Center, Taipei, Taiwan; 4 Institute of Pathology and Parasitology, National Defense Medical Center, Taipei, Taiwan; 5 Department of Pathology, Tri-Service General Hospital, Taipei, Taiwan

**Keywords:** Endometrial cancer, Immunotherapy, Microsatellite instability, Machine learning

## Abstract

Molecular classification, particularly microsatellite instability-high (MSI-H), has gained attention for immunotherapy in endometrial cancer (EC). MSI-H is associated with DNA mismatch repair defects and is a crucial treatment predictor. The NCCN guidelines recommend pembrolizumab and nivolumab for advanced or recurrent MSI-H/mismatch repair deficient (dMMR) EC. However, evaluating MSI in all cases is impractical due to time and cost constraints. To overcome this challenge, we present an effective and efficient deep learning-based model designed to accurately and rapidly assess MSI status of EC using H&E-stained whole slide images. Our framework was evaluated on a comprehensive dataset of gigapixel histopathology images of 529 patients from the Cancer Genome Atlas (TCGA). The experimental results have shown that the proposed method achieved excellent performances in assessing MSI status, obtaining remarkably high results with 96%, 94%, 93% and 100% for endometrioid carcinoma G1G2, respectively, and 87%, 84%, 81% and 94% for endometrioid carcinoma G3, in terms of F-measure, accuracy, precision and sensitivity, respectively. Furthermore, the proposed deep learning framework outperforms four state-of-the-art benchmarked methods by a significant margin (*p* < 0.001) in terms of accuracy, precision, sensitivity and F-measure, respectively. Additionally, a run time analysis demonstrates that the proposed method achieves excellent quantitative results with high efficiency in AI inference time (1.03 seconds per slide), making the proposed framework viable for practical clinical usage. These results highlight the efficacy and efficiency of the proposed model to assess MSI status of EC directly from histopathological slides.

## Introduction

Endometrial cancer (EC) comprises various histologic subtypes, collectively representing the predominant gynecologic malignancy and the second most prevalent female malignancy, following breast cancer, in developed countries^1^. Clinically, EC is stratified based on histological characteristics classified into non-aggressive and aggressive histological subtypes. In revised FIGO staging, non-aggressive histological types are composed of low-grade (grade 1; G1 and grade 2; G2) endometrioid carcinoma, representing 65% of ECs^2^. These are more likely to be diagnosed at an early stage due to the onset of symptoms (e.g., abnormal uterine bleeding or postmenopausal bleeding). These tumors are generally hormonally driven with estrogen and progesterone receptors, comprising low-grade cells, and are often preceded by precursor intraepithelial lesions with a favorable prognosis^3^. Aggressive EC includes FIGO grade 3 (G3) endometrioid carcinoma, serous, clear cell, mixed, undifferentiated carcinoma and carcinosarcoma^2^. These tumors are typically hormone-independent, with no expression of estrogen and progesterone receptors, and consist of high-grade cells, typically present at a later stage, and are associated with overexpression of HER2/neu and p53 mutations with an unfavorable prognosis^4,5^. Multiplatform molecular subtyping has been put into clinical practice as an alternative to The Cancer Genome Atlas (TCGA)-based classification of EC, which has proven to be a tool for predicting prognosis and guiding treatment^6^.

The cancer genome atlas (TCGA) research network has established a set of criteria that classify EC into four molecular subtypes, namely polymerase *ϵ* (POLE) ultra-mutated, microsatellite instability (MSI), copy-number low (CNV-L), and copy-number high (CNV-H), based on their mutation characteristics, copy-number alterations that reflect the biology of EC tumors, which may provide guidance for surgery, adjuvant therapy and disease monitoring^7,8^. Approximately 30% of primary ECs are microsatellite instability-high/hypermutated (MSI-H), and 13–30% of recurrent ECs are MSI-H or mismatch repair deficiency (dMMR)^9^. MSI-H or hypermutated subgroups have mutations in many genes owing to their generally high mutation burden^10^. PTEN, ARID1A, PIK3CA, PIK3R1 and RPL22 are all frequently mutated in the MSI subgroup of EC with extensive tumor-infiltrating lymphocytes (TILs), and immune dysregulation, immune checkpoint blockade (ICB) has been explored for targeted therapy^11^. In MSI-H or dMMR advanced EC, PD-1 inhibitors dostarlimab and pembrolizumab have shown response rates of 49% and 57%, respectively, while the PD-L1 inhibitors avelumab and durvalumab have shown response rates of 27% and 43%, respectively^9^.

Defects in the DNA MMR proteins are the primary cause of MSI^12^. The most cost-effective screening approach involving immunohistochemistry (IHC) has gained approval as a companion diagnostic test for assessing the expression of MMR proteins, namely MutL homologue 1 (MLH1), MutS homologue 2 (MSH2), MutS homologue 6 (MSH6), and postmeiotic segregation increased 2 (PMS2) expression in tumor specimens. This procedure can be readily conducted within the majority of pathology laboratories^13,14^, sensitivity is reported as 85.7% with a 91.9% specificity in a key study^15^. At the same time, other international guidelines estimate that IHC testing has a sensitivity of 94% and a specificity of 88%^16^. The algorithm for MSI testing in EC also includes a multiplex polymerase chain reaction (PCR) assay^17^. IHC-based examinations for MMR and PCR-based assessments for MSI, either individually or in combination, exhibit equivalent utility as primary screening methods in EC. These approaches demonstrate a substantial level of concordance in their results^18^. Next-generation sequencing (NGS), a high-throughput sequencing platform uses different technologies to identify genomic alterations occurring in any region of a target gene or detect covalent modifications such as methylated nucleotides; a distinctive genomic pattern associated with the MSI-H phenotype of EC was identified using a targeted NGS gene panel^8,19^. This landmark approval could be particularly beneficial for EC patients, given that 16-17% are dMMR as detected by NGS^20^. However, the current experimental screening method for testing MSI/dMMR requiring additional tumor tissue sections is laborious and time-consuming, often requiring visual inspection to categorize samples.

In recent years, deep learning (DL) researchers have shifted their focus toward addressing challenging biological problems that are not easily analyzed using traditional methods. Initiatives like TCGA have provided access to omics data, enabling the training of DL algorithms^21^. Additionally, the advancement of computing technology and the availability of whole slide images (WSIs) have facilitated the use of computer-assisted diagnostics, revolutionizing the workflow for pathologists^22,23^. To tackle these biological challenges, convolutional neural networks (CNNs) have emerged as powerful tools^22,24–33^. Supervised learning and weakly supervised learning are two commonly employed techniques in DL. Chen et al.^24^ and Coudray et al.^25^ utilized a supervised whole slide training approach to classify lung adenocarcinoma and lung squamous carcinoma, achieving promising results in terms of performance and accuracy. However, supervised learning methods rely on substantial expert or human-annotated slides for accurate model training and prediction.

To address the above-mentioned challenge, Campanella et al.^22^ introduced a weakly supervised learning approach, also called ClassicMIL, which is a combination of multiple instance learning (MIL) with Resnet34 and recurrent neural network. ClassicMIL successfully performed cancer diagnosis using WSIs in prostate cancer, skin cancer basal cell carcinoma and lymph node metastasis by selecting top-k patches without the need for pixel-level annotation. Despite its potential, this approach has some drawbacks, such as (1) the requirement of thousands of WSIs to obtain comparable performance to fully supervised classifiers, which is difficult for data curation especially in precision oncology, and (2) the possibility of selecting patches without or with minimal tumor tissues by the first stage MIL model.

Lu et al.^26^ introduced the clustering-constrained-attention multiple-instance learning (CLAM) as an attention-based pooling MIL that extracted patch features using pre-trained networks and trained a fully connected network with an attention module for non-small cell lung cancer subtype prediction. Their approach could also be adapted to weakly supervised learning without requiring pixel-level annotations. Furthermore, Lu et al.^27^ extended their CLAM-based method as Tumor origin assessment via DL (TOAD) by fusion of the patients’ gender as a co-variate data with the histopathological slide features to automatically predict metastasis status and the origin of 18 tumor types, demonstrating deep learning as a computer-aided diagnosis in identification of the site of primary origin for tumor specimens. However, the primary drawback of MIL-based methods is their tendency to consider localized regions as individual instances.

Zheng et al.^34^ introduced a Kernel Attention Transformer (KAT) for histopathology WSIs. It extracts hierarchical context information by employing cross-attention between patch-level features and spatially related kernels on WSI datasets for gastric, endometrial and lung cancer patients, demonstrating good performances without pixel-level annotations. However, Transformers often have higher computational requirements and data needs, which can pose challenges in medical image analysis, especially when resources and data are limited.

Based on the literature review, it is found that the direct assessment of MSI status in EC from hematoxylin and eosin (H&E)-stained WSIs is poorly explored. In 2023, Zhang et al.^35^ performed a small scale study with 95 patients (47 MSI-H cases and 45 MSS cases), obtaining decent results with 83%, 80% and 86% in terms of F-measure, accuracy and sensitivity, respectively. To deal with the above-mentioned challenges, we proposed a highly effective and efficient deep learning-based model to accurately and rapidly assess MSI status in EC directly from H&E-stained whole slide images, achieving remarkably high results with 96%, 94% and 100% for endometrioid carcinoma G1G2, respectively, and 87%, 84% and 94% for endometrioid carcinoma G3, in terms of F-measure, accuracy and sensitivity, respectively. Firstly, to avoid confusion in the AI training process, a smart and fast foreground localization module is built to rapidly locate foreground areas containing substantial cytoplasmic materials while eliminating regions of markers and noises. This greatly helps improve both the AI performance in training and inference and the model efficiency. Secondly, an iterative patch sampling strategy is devised to sample the representative patches of individual slides according to the patch attention scores generated from a pre-trained modified fully convolutional network of our previous efforts, which have been demonstrated successfully in tumor segmentation for various types of cancers, including diagnosis of breast cancer^36^, cervical cancer^37^ and ovarian cancer^38–40^ using histopathological slides and thyroid cancer using cytological slides^41^. This avoids the possibility of selecting patches without or with minimal tumor tissues and improves the model optimization process. Thirdly, the decision weighting model formulates the decision weights of individual representative patches based on the patch attention scores is proposed to avoid the tendency of the model to only consider localized regions or areas as individual instances. Fourthly, a weighted softmax integrated decision model is created to render a reliable slide-level decision by integration of decisions on representative patches using the associated decision weights obtained from the proposed decision weighting model. Additionally, we compared four state-of-the-art weakly supervised deep learning approaches as the benchmarked methods, which have been demonstrated to be successful in the field of computational pathology, including (1) ClassicMIL^22^ for classification of prostate cancer, skin cancer basal cell carcinoma and lymph node metastasis, (2) CLAM^26^ for subtyping of non-small cell lung cancer and renal carcinoma cancer and detection of breast cancer lymph node metastasis, (3) TOAD^27^ for predicting metastasis status and the origin of 18 tumor types and (4) KAT^34^ for cancer subtyping on gastric cancer (six subtypes), endometrium cancer (five subtypes) and lung cancer (three subtypes).

Our framework was evaluated on a comprehensive dataset of gigapixel histopathology images of 529 patients from The Cancer Genome Atlas (TCGA) in the United States. The experimental results have shown that the proposed method achieved excellent performances in assessing MSI status in different EC subtypes by obtaining remarkably high results with 96%, 94%, 93% and 100% for F-measure, accuracy, precision and sensitivity for the endometrioid carcinoma G1G2, respectively, and 87%, 84%, 81% and 94% for F-measure, accuracy, precision and sensitivity for the endometrioid carcinoma G3, respectively. Importantly, the proposed model has been demonstrated to be able to deal with imbalanced datasets (i.e. for the non-aggressive endometrial cancer dataset 30% MSI-H vs. 70% MSI-L and for the aggressive endometrial cancer dataset 41% MSI-H vs. 59% MSI-L, respectively). Moreover, the proposed method significantly outperformed four state-of-the-art deep learning approaches^22,26,27,34^ (*p* < 0.001). Additionally, the run time analysis shows that the proposed method achieves excellent quantitative results with high efficiency in AI inference with 1.03 seconds per slide using cheap GPU card, i.e. NVIDIA GTX 1080 Ti, making the proposed framework viable for practical clinical usage. We also performed run time analysis on a higher specification workstation and the result is even faster with 0.81 seconds per slide using NVIDIA GTX 2080 Ti. The above-mentioned results further validate that our proposed DL-based model could be used in MSI prediction in clinical applications, especially in healthcare settings with limited resources or low income countries.

In Results section, we present the materials and the results to compare the performance of the proposed deep learning (DL) framework with four state-of-the-art weakly supervised DL approaches^22,26,27,34^. In Discussion section, the discussion is provided. Finally, the methods are provided in Methods section.

## Results

In this study, the experiments were conducted in three parts: Firstly, we compared the proposed methods in assessing MSI status with four state-of-the-art weakly supervised deep learning approaches, which have achieved remarkably success in the field of computational pathology, including ClassicMIL^22^, CLAM^26^ TOAD^27^, and KAT^34^ using TCGA dataset. Secondly, we performed statistical analysis, employing Fisher’s Least Significant Difference (LSD) test utilizing SPSS software^42^, to compare the proposed method with the baseline approaches. Lastly, we further conducted a run-time analysis to evaluate the computational efficiency of the proposed method and four benchmarked methods.

### Patient cohorts

**Fig. 1:**
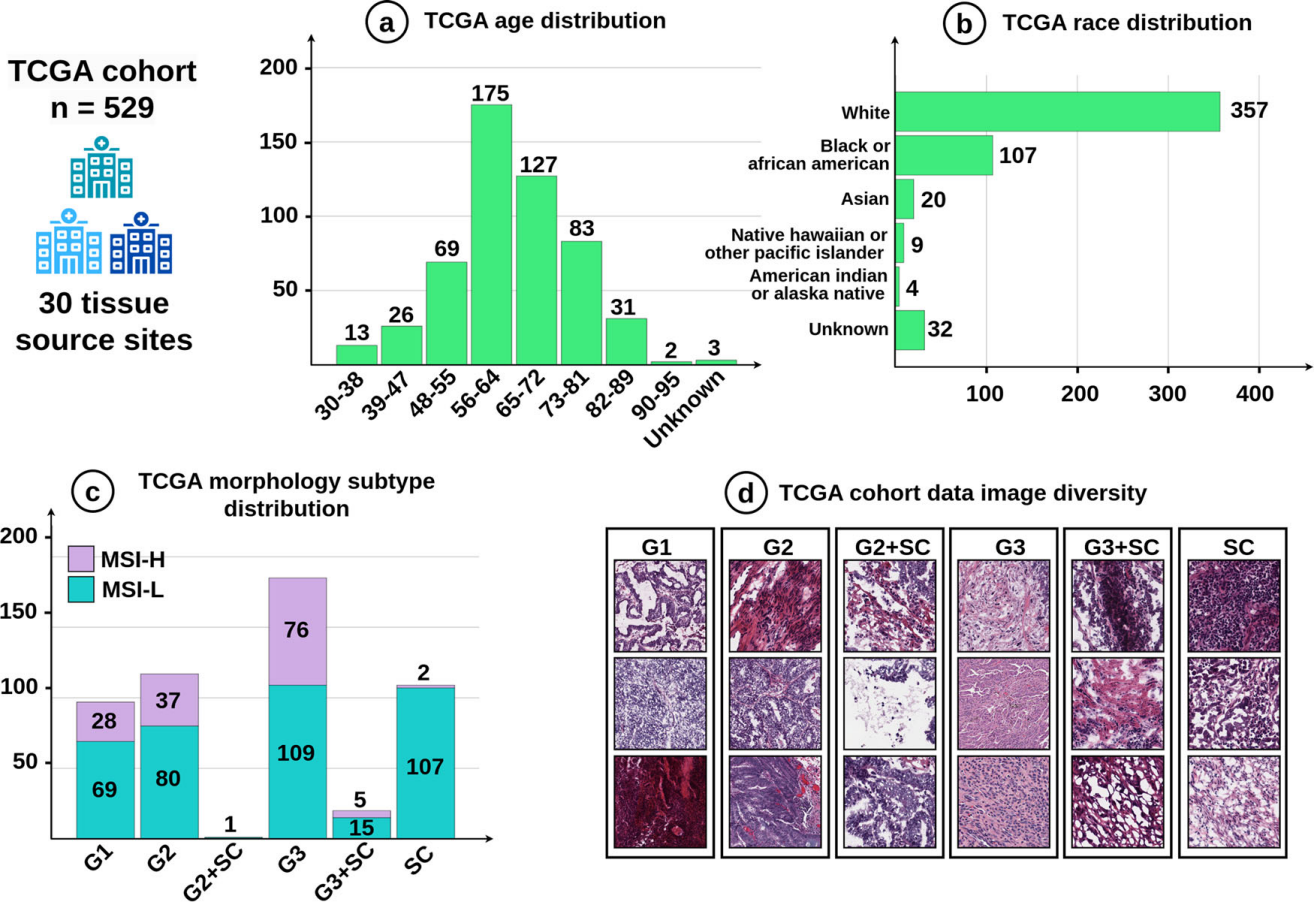
Data information. **a** TCGA age distribution. **b** TCGA race distribution. **c** TCGA morphology subtype distribution. **d** TCGA cohort data image diversity.

This study utilized anonymized H&E-stained WSIs sourced from formalin-fixed paraffin-embedded materials from the TCGA cohort. The TCGA cohort is a comprehensive collection of tissue specimens from 30 tissue source sites available in the public repositories at the National Institutes of Health, USA (https://portal.gdc.cancer.gov/), where the tissue source site is accounted for dataset sampling. The dataset consists of H&E-stained pathological WSIs with dimensions varying from 7967 to 174,281 pixels in width and 11,672 to 85,452 pixels in height. These WSIs were obtained from 529 patients diagnosed with endometrial cancer (EC), encompassing individuals aged 31–91 from over seven different races, as illustrated in Fig. 1a, b. In addition, the data contains various morphological subtypes, including endometrioid carcinoma G1 (*n* = 97), endometrioid carcinoma G2 (*n* = 117), endometrioid carcinoma G3 (*n* = 185), serous carcinoma (SC, *n* = 109), combined endometrioid carcinoma G2 and SC (*n* = 1), and combined endometrioid carcinoma G3 and SC (*n* = 20) (see Fig. 1c). Due to a significant imbalance in the number of samples between the MSI-H and MSI-L labels, including serous carcinoma (SC), combinations of endometrioid carcinoma G2 and SC, and combinations of endometrioid carcinoma G3 and SC as presented in Fig. 1c, we have chosen to exclude these subtypes from our experimental analysis. Moreover, we excluded one slide with suboptimal staining quality due to excessive thickness of the slide causing overlapping cells and less than 10% of clearly identifiable tumors by visual inspection of an experienced pathologist. In this study, we employed stratified sampling to split the cohort into patient-independent training subsets (2/3) and testing subsets (1/3), preserving the proportional representation of critical characteristics within each group.

### Microsatellite instability prediction for the TCGA dataset (NGS Label)

**Fig. 2:**
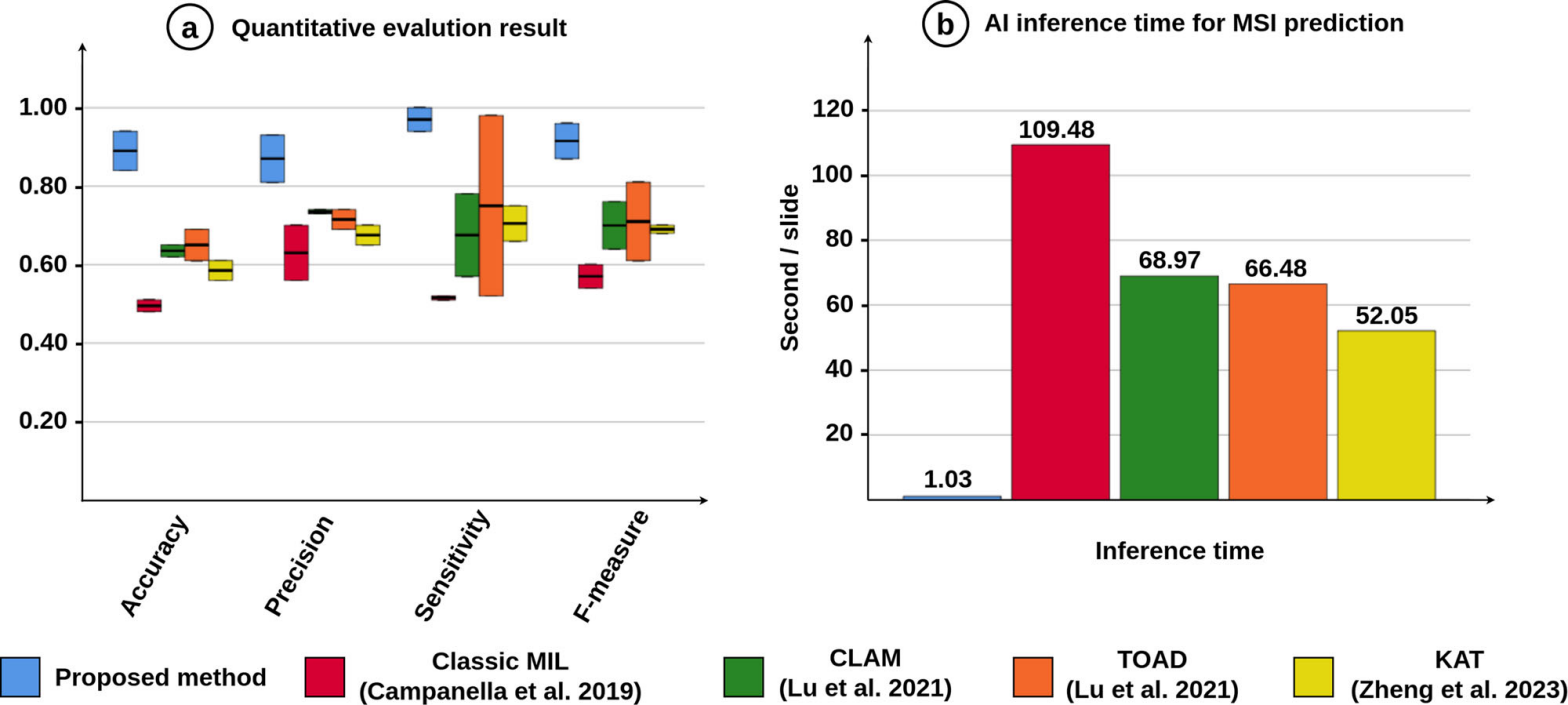
Comparison in efficacy and efficiency. **a** Box plots of the quantitative evaluation results. **b** Run time analysis for MSI prediction in comparison of the proposed DL method with ClassicMIL^22^, CLAM^26^, TOAD^27^ and KAT^34^.

Firstly, we evaluated the model performance for assessing MSI status of EC on the TCGA cohort using the NGS label. The experimental result shows that the proposed method achieved the top F1-score with 96.00%, 94.00%, 93.00% and 100.00% for F-measure, accuracy, precision and sensitivity on the endometrioid carcinoma G1G2 label, respectively. For endometrioid carcinoma G3 label, our proposed method obtained remarkably high performance with 87.00%, 84.00%, 81.00% and 94.00% for F-measure, accuracy, precision and sensitivity (see Fig. 2a) and Table 1. Moreover, the four state-of-the-art methods appear to be inferior to the proposed method with an average F1-score lower than 81.00% for endometrioid carcinoma G1G2 and 70.00% for endometrioid carcinoma G3 as presented in Fig. 2a and Table 1. The results demonstrate that the proposed DL method can predict MSI status directly from H&E slides, even for datasets with imbalanced class distributions, i.e., 65 slides of MSI-H (30.37%) and 149 slides of MSI-L class (69.63%) for endometrioid carcinoma G1G2, and 76 slides of MSI-H class (41.08%) and 109 slides of MSI-L class (58.92%) for endometrioid carcinoma G3, respectively (see Fig. 1c).

**Table 1:** Evaluation in classification of MSI status in TCGA dataset

TCGA subtypes	Method	Accuracy	Precision	Sensitivity	F-measure	F-measure Group Rank
	ClassicMIL	0.51	0.70	0.52	0.60	5
	CLAM	0.65	0.74	0.78	0.76	3
G1G2	TOAD	0.69	0.69	0.98	0.81	2
	KAT	0.56	0.70	0.66	0.68	4
	**Proposed method**	**0.94**	**0.93**	**1.00**	**0.96**	**1**
	ClassicMIL	0.48	0.56	0.51	0.54	5
	CLAM	0.62	0.73	0.57	0.64	3
G3	TOAD	0.61	0.74	0.52	0.61	4
	KAT	0.61	0.65	0.75	0.70	2
	**Proposed** **method**	**0.84**	**0.81**	**0.94**	**0.87**	**1**

The top 1 method is highlighted in the bold case.

### Statistical analysis in TCGA dataset

Moreover, we conducted a statistical analysis to compare the proposed method with the benchmarked approaches, employing Fisher’s LSD test utilizing SPSS software^42^. Compared to four state-of-the-art DL methods, the proposed method obtained significantly better results than all benchmarked approaches in terms of F-measure, accuracy, precision and sensitivity (*p* < 0.001) (see Table 2).

**Table 2:** Multiple comparisons for MSI status prediction of endometrium cancer on TCGA dataset: LSD test

LSD multiple comparison							
Measurement	(I) Method	(J) Method	Mean difference(I-J)	Std. error	Sig.	95%C.I.	
						Lower bound	Upper bound
Accuracy	Proposed method	ClassicMIL	***0.39	0.003	<0.001	0.38	0.39
		CLAM	***0.25	0.003	<0.001	0.24	0.25
		TOAD	***0.24	0.003	<0.001	0.23	0.24
		KAT	***0.29	0.003	<0.001	0.28	0.29
Precision	Proposed method	ClassicMIL	***0.24	0.005	<0.001	0.23	0.25
		CLAM	***0.12	0.005	<0.001	0.11	0.13
		TOAD	***0.14	0.005	<0.001	0.13	0.15
		KAT	***0.19	0.005	<0.001	0.18	0.20
Sensitivity	Proposed method	ClassicMIL	***0.45	0.01	<0.001	0.43	0.47
		CLAM	***0.31	0.01	<0.001	0.29	0.34
		TOAD	***0.26	0.01	<0.001	0.24	0.29
		KAT	***0.25	0.01	<0.001	0.23	0.27
F-measure	Proposed method	ClassicMIL	***0.34	0.006	<0.001	0.33	0.35
		CLAM	***0.22	0.006	<0.001	0.21	0.23
		TOAD	***0.22	0.006	<0.001	0.21	0.23
		KAT	***0.21	0.006	<0.001	0.20	0.22

The mean difference is significant at the level of *0.05, **0.01 and ***0.001.

### Run time analysis

For the runtime analysis, we evaluated the proposed method and the benchmarked methods on a workstation equipped with an NVIDIA GTX 1080 Ti GPU card and Intel Core i9-7900X CPU. Table 3 and Fig. 2 compare the model efficacy based on the inference time of the proposed method and benchmarked methods. The results show that the proposed method obtains higher efficacy with more than 50 times faster compared to the four state-of-the-art benchmarked methods in inference time, which takes approximately 1.03 s to process one WSI as illustrated in Fig. 2b. Additionally, we performed run time analysis on a higher specification workstation equipped with an NVIDIA GTX 2080 Ti GPU card and Intel Core i9-7900X CPU, and the result is even faster with 0.81 seconds per slide. These results demonstrate that the proposed method is highly efficient in terms of inference time for assessing MSI status directly using H&E-stained WSIs, making our proposed framework viable for practical clinical usage.

**Table 3:** Run time analysis in TCGA dataset

Method	Inference-time (s/slide)	CPU	GPU	Batch size
ClassicMIL	109.48	Intel Core i9-7900X	Nvidia GTX 1080 Ti	512
CLAM	68.97	Intel Core i9-7900X	Nvidia GTX 1080 Ti	512
TOAD	66.48	Intel Core i9-7900X	Nvidia GTX 1080 Ti	512
KAT	52.05	Intel Core i9-7900X	Nvidia GTX 1080 Ti	16
**Proposed method**	**1.03**	**Intel Core i9-7900X**	**Nvidia GTX 1080 Ti**	**6**
**Proposed method**	**0.81**	**Intel Core i9-7900X**	**Nvidia GTX 2080 Ti**	**6**

The top 1 method is highlighted in the bold case.

## Discussion

This study proposed an enhanced interpretable annotation-free DL model with a smart patch sampling model designed to accurately and efficiently assess MSI status in EC directly from H&E WSIs. Evaluated with the TCGA EC data, our proposed DL-based approach outperformed four state-of-the-art models^22,26,27,34^ by achieving excellent performances with 96%, 94%, 93% and 100% for F-measure, accuracy, precision, and sensitivity for endometrioid carcinoma G1G2, respectively, and 87%, 84%, 81% and 94% for F-measure, accuracy, precision, and sensitivity for endometrioid carcinoma G3, respectively. Additionally, our proposed model has shown a high efficiency with AI inference results with 1.03 seconds per WSI, making the proposed method viable for practical usage. Furthermore, the proposed DL framework outperforms four state-of-the-art benchmarked methods by a significant margin (*p* < 0.001) with respect to the accuracy, precision, sensitivity and F-measure, respectively. These results further validate that our proposed DL-based model could be used in MSI prediction in clinical applications, especially in healthcare settings where limited resources are currently prohibitive for universal molecular biology tests.

In the era of precision medicine, increasing numbers of targeted therapies have entered clinical use and undergoing trials in patients with EC. Furthermore, some biomarkers are used to select suitable patients for the related therapies. An important example of this is NGS or PCR-identified MSI-H or pathologic evaluation of DNA mismatch repair deficiency based on assessing MSI biomarkers (MLH1, PMS2, MSH2, MSH6) that are predictive of response to targeted immunotherapy, such as ICBs, including anti-PD1 antibody and anti-PDL1 antibody^43–45^. Here we show that the DL-based approach can predict MSI or MSS directly from H&E-stained WSIs of EC, which is easily performed.

The majority of individuals with EC are identified at the local stage due to uncommon, abnormal vaginal bleeding, and the 5-year survival rate is 95.0%, indicating a favorable prognosis. For medically operable patients, surgery, involving total hysterectomy and bilateral salpingo-oopherectomy with surgical staging, is the standard of treatment. Adjuvant therapy (chemotherapy or radiotherapy) is recommended based on risk factors and pathological findings^11^. In contrast, 16 out of 100 patients who got diagnosed as EC have metastatic disease with a 5-year survival rate of more than 16%, which leads disparately to disease mortality^46^. Immunotherapy is identified as an effective treatment option for EC, with significant clinical response found in certain recurrent or refractory patients^47^. MMR deficiency in MSI EC cells causes variations in the length of microsatellite sequences inside the human genome’s coding area, known as coding MSI^48^. Coding MSI is capable of creating frameshift peptides that promote oncogenesis by inactivating tumor-suppressive proteins and inducing tumor-specific immune responses^49^.

EC characterized by MSI-H or dMMR usually harbors a higher neoantigen load and increased CD3-positive, CD8-positive, and programmed death-1 (PD-1)-expressing tumor-infiltrating lymphocytes and programmed death ligand-1 (PD-L1)-expressing intraepithelial and peritumoral immune cells when compared to MSS ECs^50^. In the immune microenvironment of EC, tumor-elicited immunosuppression is mainly due to the conjugation of over-expressed PD-L1 and PD-L2 on EC cells to PD-1 receptors on tumor-infiltrating CD4+/ CD8+ T cells. ICBs can decrease the negative immunomodulation exerted by tumor cells through PD-1/PD-L1 pathways and thus restore antitumor effects of T cells^51–53^. The identification of PD-L1 expression within the tumor microenvironment has been acknowledged as a significant biomarker for determining which patients are more likely to derive benefits from immunotherapy. In the context of ECs, PD-1 expression is observed in approximately 75% of cases, while PD-L1 expression ranges from 25% to 100%, marking the highest levels of expression among gynecological malignancies. The humanized monoclonal anti-PD-1 antibody pembrolizumab is an ICB whose clinical activity has been investigated in patients with MSI-H/dMMR ECs^54,55^. A phase II trial expanded the therapeutic value of ICBs from canonical dMMR colorectal cancers to more dMMR tumor types. It drew researchers’ attention to applying ICBs in MSI EC^55^. In 2019 a phase II KEYNOTE-158 study reported that among 49 EC patients whose tumors were MSI-H/dMMR, the objective response rate (ORR) of pembrolizumab treatment was 57.1%, with a complete response rate of 16% and a partial response rate of 40%. The GARNET trial, a phase Ib study of the anti-PD-1 drug dostarlimab, found an ORR of 44.7% in dMMR EC patients and 13.4% in MMR proficient patients treated with dostarlimab^56^. Recently, nivolumab, a new PD-1 blockade, has shown significant activity in dMMR cancer^47^. Blocking PD-L1 is another effective mechanism for ICBs in EC. In 2017, the impact of a PD-L1 blockade, atezolizumab, was first assessed through a phase Ia study. Apart from atezolizumab, avelumab also emerges as a promising ICB designed to target PD-L1 in the context of EC^57^. Durvalumab, another PD-L1 inhibitor, also showed impressive effects in MSI ECs^47^.

MSI-H/dMMR could be assessed by PCR and IHC. More recently, MSI analysis by NGS was introduced. NGS can detect frequencies as low as one mutated copy among thousands of wild-type copies and elucidate multiple types of Mutational Landscapes of Tumors. NGS also has become an important aspect of precise diagnosis and treatment and tumor-targeted therapy-related driver gene detection, including in EC^58^. Before massively parallel DNA sequencing became available, MSI-H/dMMR detection mainly relied on IHC staining for the loss of one of the four MMR proteins (MLH1, PMS2, MSH2, and MSH6) expression. For PCR testing for MSI, MSI at ≥2 loci was defined as MSI-high, instability at a single locus was defined as MSI-low, and no instability at any of the loci tested was defined as MSS^59^. In larger institutions, there is a trend toward universal genomic profiling (including PCR-based MSI analysis) of newly diagnosed EC^60^. In cohort D, the determination of MSI-H/dMMR status was evaluated by PCR at a central laboratory. In cohort K, the assessment of MSI-H/dMMR status was performed either by IHC or PCR at a local laboratory.

However, heterogenous loss of MMR protein expression affects the accuracy of interpretation, with areas showing weak or no staining coexisting with areas of strong and/or diffuse staining in most cases^61–64^. PCR is inexpensive but requires skilled analysts to interpret variations in fragment length distribution. For challenging situations, results may depend on operator^65^. Although assessing MSI-H/dMMR can be determined either by PCR, NGS, or IHC, which are widely recommended methods for immunotherapy in EC^66^, a validated testing DL in predicting MSI for EC is yet to be established. Morphological features that are significantly more commonly associated with MSI-H ECs than MSS tumors include localization in the lower uterine segment, low-grade endometrioid histology, mucinous differentiation, tumor-infiltrating lymphocytes, and peritumoral lymphocytes^67–69^. Our results demonstrate that the application of DL could predict MSI status by NGS (TCGA data) based on H&E-stained WSIs in MSI-related ECs.

In tumors associated with Lynch syndrome, MSI-H or dMMR has been widely detected. In terms of prevalence, endometrial carcinoma^70^, colon adenocarcinoma^71^ and stomach adenocarcinoma^72^ rank in the top three. Following them are rectal adenocarcinoma, adrenocortical carcinoma and uterine carcinosarcoma^73^. Simultaneously, survival analysis based on TCGA shows a significant association between the expression of mismatch repair genes and the prognosis of various tumors. Tumors with MSI-H or dMMR exhibit sensitivity to ICB, particularly PD-1 and PD-L1 inhibitors. Recent studies suggest that the MMR status may serve as a candidate biomarker and predict patient responses to ICB in solid tumors, irrespective of cancer type^73^. MSI-H/dMMR also predicts the efficacy of combined ICB therapy^74^. In addition to EC, a strong clinical relationship has also been observed between the MMR status and colorectal cancer (CRC). The proportion of MSI-H/dMMR in sporadic colon cancer can be as high as 15%. Clinical-pathological variables such as proximal tumor location, advanced age (>65 years), poor differentiation, diploid DNA content, and the BRAF V600E mutation have been found to be associated with the high prevalence of MSI-H^75^. In the treatment of early-stage CRC, MSI-H or dMMR also has good prognostic predictive value without adjuvant chemotherapy. Moreover, it plays a negative predictive role for adjuvant fluorouracil-based chemotherapy in patients undergoing curative colorectal resection^76,77^. Tumor mutation burden (TMB), as compared to MSI-H/dMMR, is another promising predictor for anti-PD-1/PD-L1 immunotherapy^78^. With the rapid progress of artificial intelligence (AI), there is an opportunity to apply DL methods to pathological slide images for clinical relevance, prognosis assessment, and analysis of immune therapy indicators for different cancers, including MSI, TMB and more.

The morphological changes in WSI manifest underlying genetic changes^79^. The highly sensitive DL-based method could pre-screen patients and could trigger additional genetic testing in case of positive predictions. Even with imperfect specificity, such classifiers could speed up the diagnostic workflow and provide immediate cost-savings, especially in the context of universal MSI and dMMR testing as recommended by clinical guidelines^80^. These AI networks identified patients with specific morphological features or genetic changes based on intrinsic genetic-histologic relationships, which benefited the precision treatment.

Currently, in clinical practice, many hospitals or laboratories, due to cost considerations, use immunohistochemical staining to analyze MSH2, MSH6, MLH1 and PMS2 together or employ PCR to detect MSI. In our study, we primarily analyzed the more common subtype of EC, endometrioid carcinoma, to investigate whether the prediction of highly microsatellite unstable results obtained through NGS can be achieved using routine H&E staining. While we obtained a good predictive ability, unfortunately, due to uneven data distribution, we did not analyze serous carcinoma. In future work, we can extend our analysis to other aggressive ECs, including serous carcinoma, clear cell carcinoma, mixed carcinoma, undifferentiated carcinoma, and carcinosarcoma. Simultaneously, it can be extended to predict whether immunohistochemical staining indicators such as MSH2, MSH6, MLH1, PMS2 are deficient, PCR analysis results, molecular subtypes of EC, and predict the efficacy of immunotherapy.

The DL-based approach proposed in this study holds potential for application in predicting MSI within clinical settings, particularly in healthcare facilities where limited resources presently hinder the widespread use of molecular biology tests. This approach could substantially reduce the molecular testing load in clinical workflows, facilitating cost-effective MSI and dMMR assessments using routinely available materials. The study underscores that high MSI (MSI-H) status, as identified through NGS in EC, can be anticipated through the analysis of H&E-stained WSIs. Furthermore, MSI status can be evaluated through a DL-based AI algorithm. This development may hold noteworthy implications for the fields of diagnosis, prognosis, and the prediction of drug responses.

## Methods

### Ethics approval and consent to participate

The protocol of this study was approved by the Ethics Committee of the Institutional Review Board of the Tri-Service General Hospital (TSGHIRB No.1-107-05-171 and No. B202005070). Informed consents were waived by the Committee due to the retrospective and anonymous nature.

### Proposed DL-based framework

**Fig. 3:**
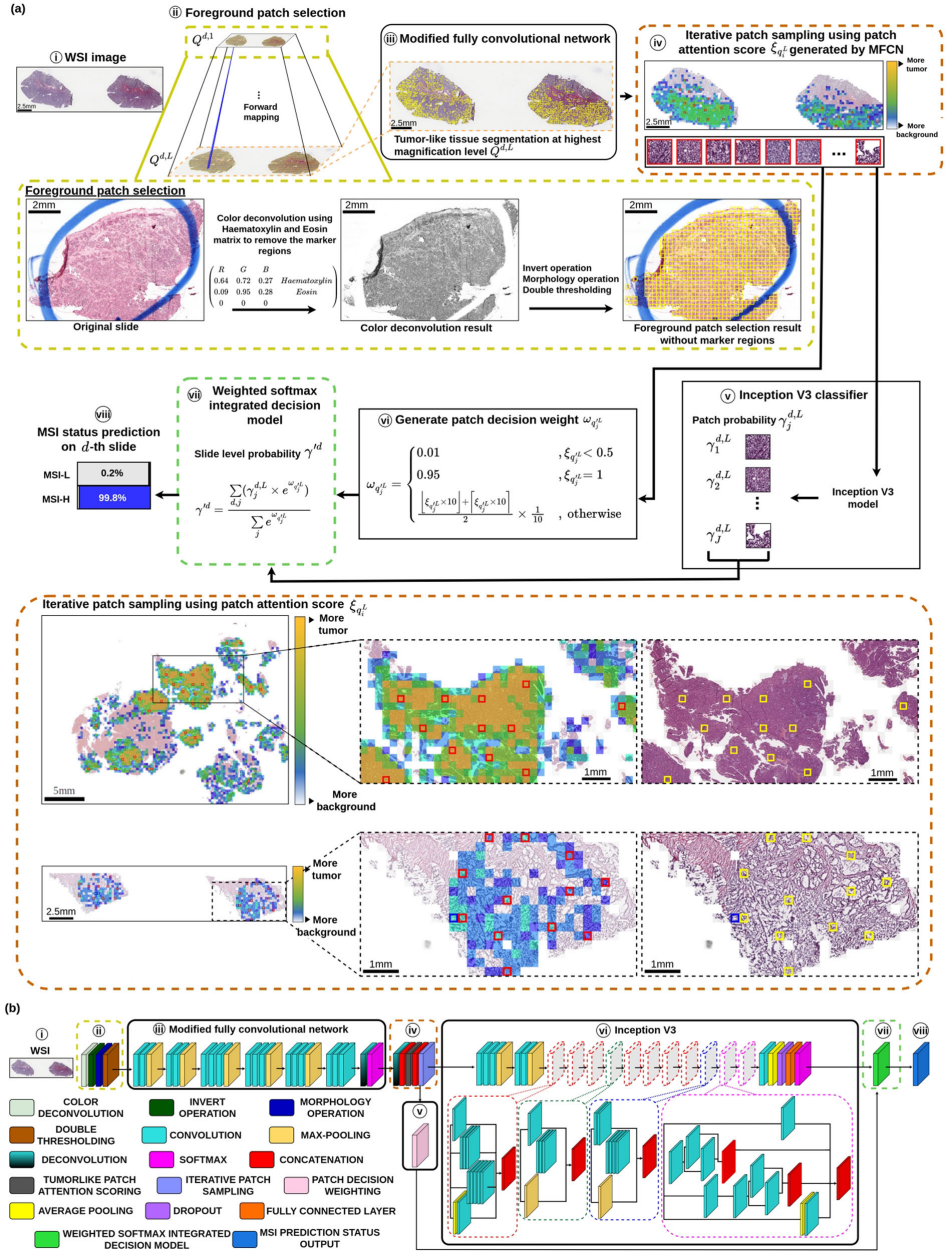
Flowchart and network architecture of the proposed method. **a** Flowchart of the proposed DL method. (i) WSIs in multi-resolution pyramid tile-based structure ${\left\{{{{{\rm{Q}}}}}^{l}\right\}}_{l = 1}^{L}$ are fed into (ii) the proposed foreground patch selection (FPS) model to rapidly locate high-resolution foreground patches $\left\{{{{{\bf{u}}}}}_{i}^{d,L}\right\}$ without marker regions annotated by medical experts. Then, (iii) the weakly supervised tumor-like tissue segmentation model Ψ_tumor_ applies to the selected foreground patches $\left\{{{{{\bf{u}}}}}_{i}^{d,L}\right\}$ to further generate the tumor-like patch attention score ${{{{\boldsymbol{\xi }}}}}_{{q}_{j}^{{\prime} L}}$. Next, (iv) the proposed iterative patch sampling (IPS) method samples representative patches $\left\{{{{{\bf{q}}}}}_{j}^{{\prime} L}\right\}$ with high attention score ${{{{\boldsymbol{\xi }}}}}_{{q}_{j}^{{\prime} L}}$. Afterwards, (v) the individual patch probability ${{{{\boldsymbol{\gamma }}}}}_{j}^{d,L}$ of the representative patch ${{{{\bf{q}}}}}_{j}^{{\prime} L}$ is obtained using InceptionV3 classifier, while (vi) the individual patch decision weight ${{{{\boldsymbol{\omega }}}}}_{{q}_{j}^{{\prime} L}}$ of the representative patch ${{{{\bf{q}}}}}_{j}^{{\prime} L}$ is computed. Subsequently, (vii) the proposed weighted softmax integrated decision (WSID) model produces a reliable and robust slide level probability ${\gamma }^{{\prime} d}$. (viii) Finally, the MSI status prediction ${D}_{MSI}^{d}$ of the *d*-th patient is generated. **b** The detailed networks of the proposed DL method.

**Fig. 4:**
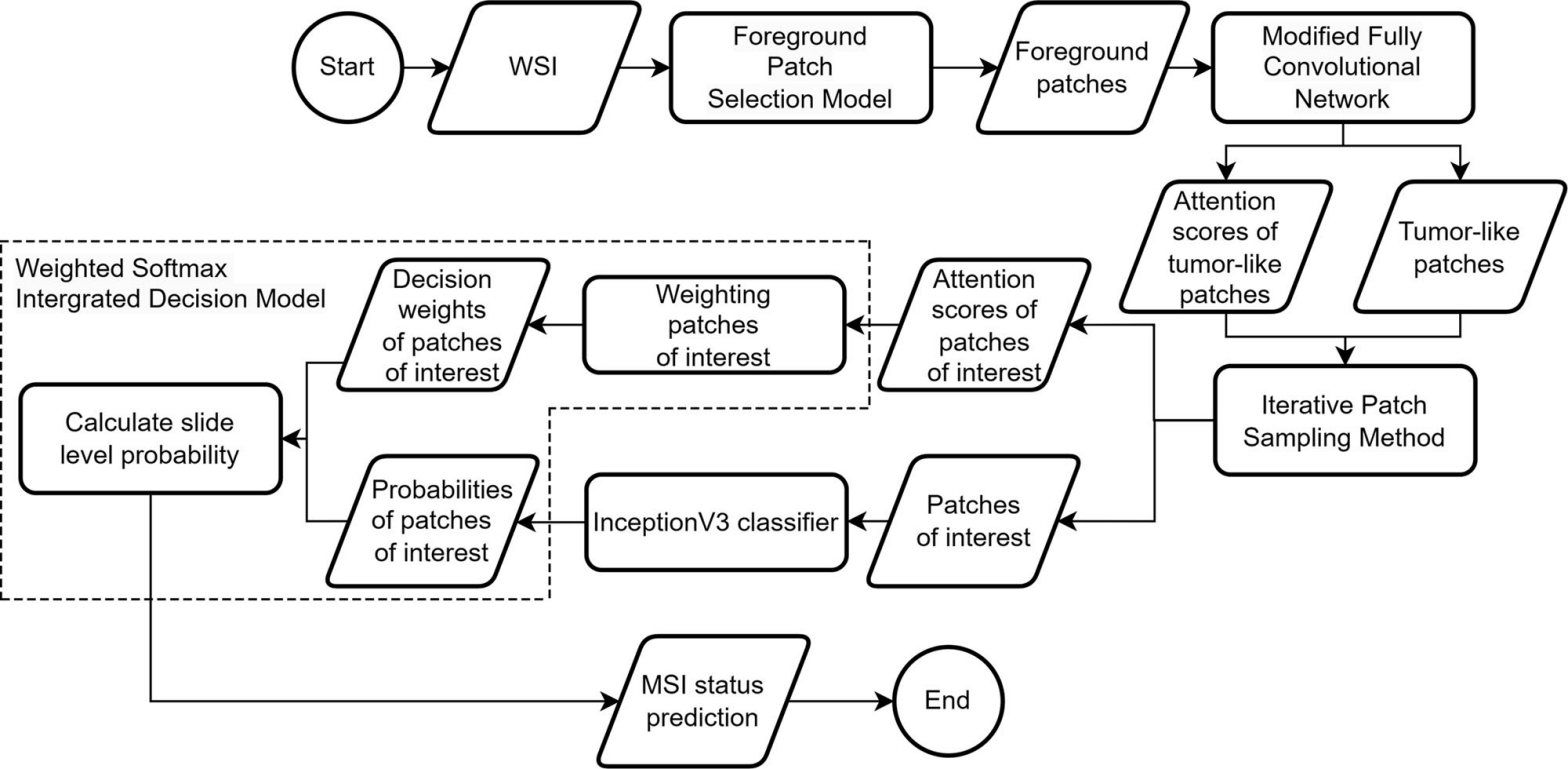
Workflow diagram of the proposed DL method. Firstly, a WSI in multi-resolution pyramid tile-based structure is fed into the proposed foreground patch selection (FPS) model to rapidly locate high-resolution foreground patches without marker regions annotated by medical experts. Then, the modified fully convolutional network model is applied to the selected foreground patches to further generate the tumor-like patch attention scores. Next, the proposed iterative patch sampling (IPS) method samples representative patches with high attention scores. Afterwards, individual patch probabilities of the representative patches are obtained using InceptionV3 classifier, while the individual patch decision weights of the representative patches are computed. Subsequently, the proposed weighted softmax integrated decision (WSID) model produces a reliable and robust slide level probability. Finally, the MSI status prediction is generated.

This study introduced a highly effective and efficient deep learning-based model to accurately and rapidly assess MSI status in EC using H&E-stained WSIs. All the slides were directly downloaded from the TCGA platform, and we did not apply any pre-processing techniques like stain normalization or data augmentation. Firstly, a foreground patch selection module is built to eliminate the clinician markers and to mitigate the undesired noises, rapidly extracting non-overlapping foreground patches (see Fig. 3a(ii)). Secondly, an iterative patch sampling strategy is devised to sample the representative patches of individual slides according to the patch attention scores generated from a pre-trained modified fully convolutional network of our previous efforts, which have been demonstrated successfully in tumor segmentation for various types of cancers, including diagnosis of breast cancer^36^, cervical cancer^37^ and ovarian cancer^38–40^ using histopathological slides and thyroid cancer diagnosis using cytological slides^41^ (Fig. 3a(iv)). Thirdly, the sampled representative patches of each slide are then processed by an Inception V3 classifier to generate patch-based probabilities (Fig. 3a(v)), and in the meantime, we computed the decision weight of individual patches using the patch attention score. Lastly, a weighted softmax integrated decision model is constructed for producing the final MSI status prediction by (see Fig. 3a(vii)). The workflow diagram in this study is provided in Fig. 4.

### Foreground patch selection (FPS) model

Given WSIs in multi-resolution pyramid tile-based structure ${\left\{{{{{\rm{Q}}}}}^{l}\right\}}_{l = 1}^{L}$, where *l* denotes the layer in the multi-resolution pyramid data structure. We devised the proposed foreground patch selection (FPS) model, a smart and fast foreground localization module is built to rapidly locate foreground areas containing substantial cytoplasmic materials while eliminating regions of markers and noises. This greatly helps improve both the AI performance in training and inference and the model efficiency (see Fig. 3a(ii)). Furthermore, FPS selects foreground region at the lowest magnification level U^*d*,1^ and performs forward mapping to the raw data at highest magnification level Q^*d*,*L*^ to acquire high-resolution foreground patches $\left\{{{{{\bf{u}}}}}_{i}^{d,L}\right\}$ formatted as patch-based format with size ι × ι where *d*, *i* and ι represent the patient id, patch id and patch size, respectively (where ι = 512 in this study).

In conventional H&E staining, Hematoxylin induces the black staining of nuclei, and on the other hand, Eosin induces the red or pink staining of cytoplasm. Our previous studies^81–83^ successfully employed color deconvolution techniques to establish robust image registration methods for diverse applications, including X-ray and biological microscopic tissue images, which usually have complex deformation challenges. In this study, we employ color deconvolution to devise the FPS model, enabling the extraction of the foreground region containing substantial cytoplasmic information by extracting the independent eosin channel from individual H&E WSIs.

Within the RGB color space, each color is represented as $\vec{m}\equiv \left({m}_{1},{m}_{2},{m}_{3}\right)\equiv \left(r,g,b\right)$, where $\left(r,g,b\right)$ denote the red, green, and black components, respectively. Additive color mixing is visualized as the vector addition of RGB components. To conceptualize the colors in an image as the vector addition of desired (Γ) and undesired (Π) components to a background color (Λ), new vectors can be defined as follows.

1 $$\vec{\rho }\equiv \vec{\Lambda \Pi }$$

2 $$\vec{\eta }\equiv \vec{\Lambda \Gamma }$$

3 $$\vec{t}\equiv \vec{\rho }\times \vec{\eta }$$

where $\vec{t}$ is perpendicular to $\vec{\rho }$ and $\vec{\eta }$; $\vec{t},\vec{\rho },\vec{\eta }$ span the 3D space; $\vec{\Lambda \Pi }$ and $\vec{\Lambda \Gamma }$ represent alternative unit vectors corresponding to the undesired and desired colors, respectively.

Subsequently, color $\vec{m}$ can be transformed to the new unit vectors.

4 $$\vec{m}=r\cdot \vec{r}+g\cdot \vec{g}+b\cdot \vec{b}=\rho \cdot \vec{\rho }+\eta \cdot \vec{\eta }+t\cdot \vec{t}+\vec{\varrho }$$

where $\vec{\varrho }\equiv \vec{O\Lambda };O$ represents the origin point within the RGB 3D space; $\vec{O\Lambda }$ is a vector.

By setting *ρ* = 0, the undesired component is effectively eliminated, resulting in the new color $\vec{{m}^{{\prime} }}=\eta \cdot \vec{\eta }+t\cdot \vec{t}+\vec{\varrho }$. In the context of three color channels, this color system can be represented as a matrix. Each row corresponds to a specific stain, while each column represents the optical density (*OD*) as observed by the red, green, and black channels for each stain.

5 $$M=\left(\begin{array}{ccc}{m}_{11}&{m}_{12}&{m}_{13}\\ {m}_{21}&{m}_{22}&{m}_{23}\\ {m}_{31}&{m}_{32}&{m}_{33}\end{array}\right)$$

For normalization, each OD vector is divided by its total length, such that $\left(\widehat{{m}_{11}}=\frac{{m}_{11}}{\sqrt{{m}_{11}^{2}+{m}_{12}^{2}+{m}_{13}^{2}}},\widehat{{m}_{21}}=\frac{{m}_{21}}{\sqrt{{m}_{21}^{2}+{m}_{22}^{2}+{m}_{23}^{2}}}\,{{{\rm{and}}}}\,\widehat{{m}_{31}}=\frac{{m}_{31}}{\sqrt{{m}_{31}^{2}+{m}_{32}^{2}+{m}_{33}^{2}}}\right)$. In this study, we define the normalized OD matrix, denoted as $\widehat{M}$, which describes the color system for orthonormal transformation, as follows:

6 $$\widehat{M}=\left(\begin{array}{cccc}R&G&B&\\ 0.6442&0.7166&0.2668&Haematoxylin\\ 0.0928&0.9541&0.2831&Eosin\\ 0&0&0&\end{array}\right)$$

When ϒ represents the 3 × 1 vector indicating the stain amounts at the specific pixel, it is feasible to denote the vector representing the detected OD levels at that pixel as $\varphi =\widehat{\Upsilon M}$. Consequently, multiplying the OD image by the inverse of the OD matrix yields an orthogonal representation of the stains composing the image $\left(\Upsilon =\widehat{{M}^{-1}}\varphi \right)$. Subsequently, we extract the image features related to the eosin channel to do further foreground patch selection approaches. Afterward, we apply dual-thresholding followed by morphological operations to extract the foreground regions while excluding marking areas annotated by medical experts (see Fig. 3b(ii)).

Lastly, the high-resolution foreground patches $\{{{{{\rm{u}}}}}_{i}^{d,L}\}$ can be formulated as follows.

7 $${{{{\bf{u}}}}}_{i}^{d,L}={{{{\rm{U}}}}}^{d,1}(x,y)\to {{{{\rm{ROI}}}}}_{(x,y)\in {{{{\rm{Q}}}}}^{d,L}}:\langle x\times {2}^{L},y\times {2}^{L},\iota ,\iota \rangle$$

where ι, U^*d*,1^ and Q^*d*,*L*^ denote the patch size, the selected foreground region at the lowest magnification level and raw data at the highest magnification level, respectively.

### Modified fully convolutional network

In this study, we adopted the pre-trained modified fully convolutional network (MFCN) of our previous efforts, which have been demonstrated successfully in tumor segmentation for various types of cancers, including diagnosis of breast cancer^36^, cervical cancer^37^ and ovarian cancer^38–40^ using histopathological slides and thyroid cancer using cytological slides^41^. Moreover, in the Automatic Cancer Detection and Classification in Whole Slide Lung Histopathology Challenge 2019 (the ACDC@LungHP Challenge 2019)^84^, the MFCN ranks 1st for the single model and 3rd overall for all models in terms of model sensitivity. Importantly, the ACDC@LungHP challenge 2019 results also show that the pre-trained MFCN does not require any preprocessing or label refinement (see Table 4.

**Table 4:** Comparison of Top 10 teams for IEEE Automatic Cancer Detection and Classification in Whole Slide Lung Histopathology Challenge 2019

	Deep learning algorithm (Top 10 methods of 391 qualified international teams)				Performance evaluation		
	Team	Label Refine	Architecture	Preprocessing	Accuracy	Sensitivity	Specificity
Multi Model	PATECH	v	DenseNet & dilation block with Unet	Color normalization; Otsu to refine label	**0.951**	**0.905**	0.953
	Byungje Lee	v	ResNet50 & DeepLab V3+	Multi data augmentations; Otsu to refine label	**0.951**	**0.863**	**0.961**
	Turbolag	–	U-Net & ConvCRF	Multi-resolution training data	**0.946**	0.847	**0.959**
	ArontierHYY	v	Mdrn80 +DenseNet & ResNet	Tile labeling strategy	0.929	0.856	0.940
	Newhyun00	v	DenseNet103	Select clean labels	0.931	0.820	0.949
Single Model	CMIAS	v	DenseNet121 & FCN	Locate the tissue regions by a bounding box	0.938	0.800	**0.957**
	Jorey	v	IncRes+ACF & CRF	Otsu to refine labels; Divided into 3 classes (tumor; normal; mix) and mix Mix file into other classes	0.937	0.815	0.951
	**Our pre-trained MFCN**	–	FCN	None	0.923	**0.860**	0.928
	Skyuser	–	ResNet18	Multi data augmentations;	0.932	0.767	0.956
	Vahid	–	Small-FCN-521	None	0.921	0.846	0.932

The top 3 methods are shown in bold format. Information reported in this table could be referred to Li et al.

The MFCN weakly supervised tumor-like tissue segmentation model Ψ_tumor_ from our previous work is applied to the selected foreground patches $\left\{{{{{\bf{u}}}}}_{i}^{d,L}\right\}$ to further generate the tumor-like patch attention score utilizing tissue pixel probabilities ${\left\{{p}_{i}^{d,L}(x,y)\right\}}^{h}$ as presented as follows (see Fig. 3a(iii)).

8 $${\left\{{p}_{i}^{d,L}(x,y)\right\}}^{h}={\Psi }_{{{{\rm{tumor}}}}}\,\left\{{u}_{i}^{d,L}(x,y)\right\}$$

where *h* ∈ {0, … , *H*}; *h* = 0, 1, 2 denotes the background class, non-tumor-like tissue class and tumor-like tissue class, respectively. Figure 3b(iii) shows the detailed architecture of our modified fully convolutional network.

A data cleaning module is formulated in Eq. (9) to extract the tumor-like tissue information and suppress the rest of the information, producing clean tumor-like data $\left\{{c}_{i}^{d,L}(x,y)\right\}$ as defined as follows.

9 $${c}_{i}^{d,L}(x,y)=\left\{\begin{array}{ll}{u}_{i}^{d,L}(x,y)\quad {{{{,}}\; {\rm{arg}}}}{\max }_{h}\,{\left\{{p}_{i}^{d,L}(x,y)\right\}}^{h} \,>\, 1\\ {{\emptyset}}\quad\quad\quad\quad\;\; ,\,{{{\rm{otherwise}}}}\end{array}\right.$$

Then, the tumor-like patch attention score ${{{{\boldsymbol{\xi }}}}}_{{c}_{i}^{L}}$ is computed based on clean tumor-like patches $\left\{{{{{\boldsymbol{c}}}}}_{i}^{L}\right\}$ on the highest magnification level as follows.

10 $${{{{\boldsymbol{\xi }}}}}_{{c}_{i}^{L}}=\frac{{{{\rm{card}}}}({{{{\bf{c}}}}}_{i}^{L})}{{{{\rm{card}}}}({{{{\bf{u}}}}}_{i}^{L})}$$

where ${{{\rm{card}}}}({{{{\bf{c}}}}}_{i}^{L})$ and ${{{\rm{card}}}}({{{{\bf{u}}}}}_{i}^{L})$ denote the cardinality of tumor-like patch set and the cardinality of original patch set, respectively.

Furthermore, to avoid confusion or distraction in AI training and inference, a data validation module is built to guarantee that each valid tumor-like patch sample ${{{{\rm{q}}}}}_{i}^{L}$ contains a minimum *α* level of tumor-like information as described as follows.

11 $${{{{\bf{q}}}}}_{i}^{L}=\left\{\begin{array}{ll}{{{{\bf{c}}}}}_{i}^{L}\quad ,{{{{\boldsymbol{\xi }}}}}_{{c}_{i}^{L}} \,>\, \alpha \\ {{\emptyset}}\quad\;\; ,\,{{{\rm{otherwise}}}}\end{array}\right.$$

where *α* is set as 0.1 in this study.

### Iterative patch sampling (IPS) method

We proposed the iterative patch sampling (IPS) method to extract and locate representative valid tumor-like patch ${{{{\rm{q}}}}}_{i}^{L}$ with high attention score ${{{{\boldsymbol{\xi }}}}}_{{q}_{j}^{{\prime} L}}$. This avoids the possibility of selecting patches without or with minimal tumor tissues and improves the model optimization process (see Fig. 3a(iv)). Next, the IPS method will iteratively sample the representative patches $\left\{{{{{\bf{q}}}}}_{j}^{{\prime} L}\right\}$ with a specified distance Δ which is formulated as follows (where Δ = 3 in this study).

12 $${{{{\bf{q}}}}}_{j}^{{\prime} L}=\left\{\begin{array}{ll}\mathop{{{{\rm{argmax}}}}}\limits_{{{{{{\bf{q}}}}}_{i}}^{{{{\boldsymbol{L}}}}}}\;{{{{\boldsymbol{\xi }}}}}_{{{{{\bf{q}}}}}_{i}}^{{{{\boldsymbol{L}}}}}\qquad\qquad\qquad\qquad\qquad\qquad\qquad\quad\;\; ,\,j=1\\ \mathop{{{{\rm{argmax}}}}}\limits_{{{{{{\bf{q}}}}}_{i}}^{{{{\boldsymbol{L}}}}}}\;{{{{\boldsymbol{\xi }}}}}_{{{{{\bf{q}}}}}_{i}}^{{{{\boldsymbol{L}}}}}\bigg| | {(x,y)}_{{{{{\bf{q}}}}}_{i}}^{{{{\boldsymbol{L}}}}}-{\{{(x,y)}_{{{{{\bf{q}}}}}_{k}^{{\prime} }}{{{\boldsymbol{L}}}}\}}_{k = 1}^{j-1}| \ge \Delta \quad ,\,{{{\rm{otherwise}}}}\end{array}\right.$$

### Weighted softmax integrated decision (WSID) model

Lastly, we proposed the weighted softmax integrated decision (WSID) model to render a reliable slide-level decision by integration of decisions on representative patches using the associated decision weights obtained from the proposed decision weighting model. The proposed WSID avoids the tendency of the model to only consider localized regions or areas as individual instances. The WSID model calculates slide level probability ${\gamma }^{{\prime} d}$ as formulated as follows (see Fig. 3a(vii)).

13 $${\gamma }^{{\prime} d}=\frac{{\sum}_{d,j}({\gamma }_{j}^{d,L}\times {e}^{{\omega }_{{q}_{j}^{{\prime} L}}})}{{\sum}_{j}{e}^{{\omega }_{{q}_{j}^{{\prime} L}}}}$$

where ${\gamma }_{j}^{d,L}$ is the patch probability of the representative patch ${{{{\bf{q}}}}}_{j}^{{\prime} L}$, which can be computed by Eq. (14), and ${\omega }_{{q}_{j}^{{\prime} L}}$ denotes individual patch decision weight, as formulated in Eq. (15).

The individual patch probability ${{{{\boldsymbol{\gamma }}}}}_{j}^{d,L}$ of the representative patch ${{{{\bf{q}}}}}_{j}^{{\prime} L}$ of the *d*-th patient is obtained using the InceptionV3 classifier Ψ_*classifier*_ as shown in Eq. (14) (see Fig. 3a(v)).

14 $${\gamma }_{j}^{d,L}={\Psi }_{classifier}({{{{\bf{q}}}}}_{j}^{{\prime} d,L})$$

Additionally, the WSID model computes individual patch decision weight ${{{{\boldsymbol{\omega }}}}}_{{q}_{j}^{{\prime} L}}$ based on the tumor-like patch attention score ${{{{\boldsymbol{\xi }}}}}_{{q}_{j}^{{\prime} L}}$ of the representative tumor-like patch ${{{{\bf{q}}}}}_{j}^{{\prime} L}$ as described below (see Fig. 3a(vi)).

15 $${{{{\boldsymbol{\omega }}}}}_{{q}_{j}^{{\prime} L}}=\left\{\begin{array}{ll}0.01\qquad\qquad\qquad\qquad\quad\;\; ,\,{\xi }_{{q}_{j}^{{\prime} L}} \,<\, 0.5\\ 0.95\qquad\qquad\qquad\qquad\quad\;\;,\,{\xi }_{{q}_{j}^{{\prime} L}}=1\\ \frac{\lfloor {\xi }_{{q}_{j}^{{\prime} L}}\times 10\rfloor +\lceil {\xi }_{{q}_{j}^{{\prime} L}}\times 10\rceil }{2}\times \frac{1}{10}\quad\;,\,{{{\rm{otherwise}}}}\end{array}\right.$$

Finally, the MSI status prediction ${D}_{MSI}^{d}$ of the *d*-th patient is computed as follows, where *δ* is set to 0.5 (see Fig. 3a(viii)).

16 $${D}_{MSI}^{d}=\left\{\begin{array}{ll}{{{\rm{MSI}}}}{{{\rm{High}}}}\quad,{\gamma }^{{\prime} d} \,<\, \delta \\ {{{\rm{MSI}}}}{{{\rm{Low}}}}\quad\;,{\gamma }^{{\prime} d}\ge \delta \end{array}\right.$$

### Implementation details

In the training process, we utilized InceptionV3 framework^85^ as a baseline model and used the root mean square propagation (RMSProp) optimizer. The models were trained with a batch size of six applying cross-entropy loss. The proposed model is then refined with an initial learning rate, weight decay and RMS decay of 3 × 10^−3^, 3 × 10^−4^ and 0.9, respectively. Furthermore, we developed and trained the models for the benchmarked approaches using the original settings from the corresponding literature (Table 5).

**Table 5:** Links to the source code of all benchmarked methods and the proposed method

Publication	Method	Code Link
Campanella et al.^22^	ClassicMIL	https://github.com/MSKCC-Computational-Pathology/MIL-nature-medicine-2019
Lu et al.^26^	CLAM	https://github.com/mahmoodlab/CLAM
Lu et al.^27^	TOAD	https://github.com/mahmoodlab/TOAD
Zheng et al.^34^	KAT	https://github.com/Zhengyushan/kat
This study	Proposed method	https://github.com/cwwang1979/DL-Can-Predict-Key-Biomarkers-for-MSI-from-the-HE-Stained-WSIs-in-non-aggressive-and-aggressive-EC

## Data Availability

The data that support the findings of this study are publicly available online through the TCGA’s Genomic Data Commons (https://portal.gdc.cancer.gov/) with the project ID as TCGA-UCEC. The exact case IDs and associated labels can be accessed on the (https://docs.google.com/spreadsheets/d/1e94eCzLOorruO3Htv1Kg8FFvZGJeSpS5uMx1-x-U1Kc/edit?usp=sharing). Full clinical information could be downloaded from (https://www.cbioportal.org/study/summary?id=ucec_tcga_pan_can_atlas_2018 (Uterine Corpus Endometrial Carcinoma (TCGA, PanCancer Atlas)).

## Code availability

The proposed DL model was deployed using the Caffe framework in Python and the program code has been made publicly accessible on GitHub (https://github.com/cwwang1979/Deep-Learning-to-Assess-Microsatellite-Instability-Directly-from-Histopathological-Whole-Slide-Image) (Please use the password to unzip the program file: *@xdrgbhu_cwlab*). Table 5 also provides links to the source code of four benchmarked frameworks and the proposed method.
